# Qualitative study of walking football participation and wellbeing among caregivers of people living with dementia

**DOI:** 10.1038/s41598-026-49922-0

**Published:** 2026-04-24

**Authors:** Oluwakemi Odufuwa, Ivelina Tsocheva, Bernice Njita, David Hewson¹

**Affiliations:** Institute for Health Research, University of Bedfordshire, Luton, UK

**Keywords:** Dementia caregiving, Social prescribing, Community sport, Caregiver wellbeing, Relational care, Health care, Health humanities, Medical humanities, Psychology, Psychology

## Abstract

Walking Football (WF) is a modified version of football played at walking pace that was developed to increase physical activity levels in people who do not engage in traditional forms of physical activity. The focus on walking football was initially on people aged 50 and over with conditions such as diabetes, but more recently WF has been studied in people living with dementia. However, research in this area is still limited, particularly when the benefits of WF are explored from the perspective of the caregivers of people living with dementia (PLWD). This study aims to explore the perceptions and experiences of caregivers in terms of the influence of WF in relieving the caregivers’ burden and the quality of life (QOL). The study uses a qualitative approach, with semi-structured interviews held with caregivers of PLWD who participate in WF. Thematic analysis identified five themes relating to caregivers’ experiences: caregiver burden, social interaction, emotional and mental wellbeing, embodied activity and functional engagement, and quality of life. Three themes were identified for people living with dementia: memory recollection, physical activity, and community belonging and peer support. The study highlights that participation in WF sessions may reduce perceived caregiving strain, improve social support, and contribute to caregivers’ overall wellbeing and QOL. The findings suggest that WF may function as a shared psychosocial activity, offering caregivers opportunities for respite, social interaction, and physical activity while supporting the wellbeing of people living with dementia.

## Introduction

The number of people worldwide who are living with dementia was estimated at 55 million in 2019, with this number predicted to rise to close to 139 million people by 2050 (World Health Organization,^1^. A key risk factor associated with developing dementia is physical inactivity^2^, while physical activity (PA) is recommended for people living with dementia due to numerous health benefits^3^. The best form of PA for people living with dementia is yet to be determined, with positive results have been reported for a range of exercises, including resistance and aerobic training^4^. Participating in sporting or social activities have also been shown to provide benefits both people with dementia and their caregivers^5,6^. Given the range of beneficial physical activities, the importance of adherence has been noted, with slower cognitive decline associated with increased adherence^4^. The inclusion of opportunities to socialise could increase adherence to PA^7^, while cultural acceptability is also important^8^.

Dementia also has an impact on the mental and physical wellbeing of family members and caregivers^9^. Caregivers have a lower quality of life resulting from their caregiving role^10^, while they are also more vulnerable to developing their own health problems^11^. In one review, it was reported that caregivers may experience significant restrictions on personal autonomy and social participation due to caregiving responsibilities^12^. Research has shown that caregivers getting involved in social activities is key to both mental and physical wellbeing^13^, while participation in physical activity with the person they are caring for is also beneficial^14^.

The most popular sport in the world is football, which can combine PA with social activity and has been shown to improve metabolic, cardiovascular, and physical fitness for all age groups^15,16^. However, playing football when older can be difficult as speed of movement is important^17^. A solution was developed in 2011 in the UK, when a walking modification of football called walking football (WF) was created^18^ that was specifically designed for older people and forbids players from running and places limits on player-to-player physical contact (The Walking Football Association,^19^. Several studies on WF have shown benefits for people with a range of conditions such as type II diabetes and hypertension^20,21^. Recent studies have also shown that WF is enjoyable, with participants reaching a moderate level of PA that could be sufficient to protect against non-communicable diseases^17,22^.

There have been limited studies of WF for people living with dementia. An initial study included a range of different activities, one of which was WF^23^. A subsequent study focused exclusively on WF for people with dementia and investigated the social influence of the programme^24^, while more recently the social, mental and physical benefits in people with dementia from WF were also explored^25^. These studies reported that the game provided a form of social interaction for PLWD, while their caregivers were able to take time off the caring role or had the time to socially associate with other family members and carers of participants while the game was going on. However, these studies did not focus on the wellbeing and quality of life of the carers and family members, as well as the benefits of walking football for them. Carers were asked in the qualitative interviews about their perceptions concerning the PLWD they were caring for, not about their own experiences.

The conceptual framework of this study draws on relational models of dementia care, particularly Kitwood’s concept of personhood, which emphasises the importance of maintaining identity and meaningful relationships in dementia care^26,27^. Activities that enable shared participation may support relational continuity between caregivers and people living with dementia. Additionally, literature on caregiver burden and caregiver gain highlights that caregiving experiences include both strain and positive meaning-making. Walking football may represent a context in which both dimensions coexist, allowing caregivers to experience shared enjoyment, identity reconstruction, and social belonging.

The focus of this study is specifically on caregivers’ perspectives and aims to explore their perception and experiences in terms of the influence of WF on the caregivers, including caregiver burden and quality of life. While previous studies have explored the experiences of people living with dementia participating in physical activity programmes, fewer studies have examined these activities from the perspective of caregivers. Caregivers play a central role in enabling participation in community-based activities and are directly affected by the emotional, social, and logistical demands of dementia care. Understanding caregivers’ experiences provides insight into how interventions such as walking football may influence caregiver wellbeing, relational dynamics, and engagement with supportive community activities. Exploring these perspectives, therefore, contributes to a more holistic understanding of the influence of sport-based dementia interventions.

## Methods

A qualitative research design was adopted, with three of the researchers (OO, BN, and DH) actively taking part in some of the WF sessions, which enabled them to understand caregivers’ perceptions through informal conversations before any formal data collection took place. The WF sessions were organised by a community dementia support organisation in the United Kingdom that provides support and activities for people with dementia and their caregivers. The sessions took place at a sports centre every Wednesday, with the football lasting 60 min, including a warm-up, followed by 30–60 min for refreshments and socialisation.

Six participants were recruited, comprising of two spousal carers, one family carer, one volunteer who had been a spousal carer, and two volunteers. Volunteers were involved in facilitating or supporting the walking football sessions. These individuals were included because of their close and sustained interaction with both caregivers and people living with dementia, which provided complementary perspectives on caregiver experiences and the social dynamics of the sessions. One volunteer also had prior lived experience as a spousal caregiver, further enriching the dataset. While the sample size was limited to six participants, this is consistent with qualitative best practices aimed at depth over breadth. As recommended by qualitative researchers, data saturation may be reached with small, purposefully selected samples in focused studies^28^. Given the exploratory nature of this study and the focused participant group, the aim was to generate in-depth, context-specific insights rather than achieve broad generalisability or transferability. No substantially new themes emerged after the final interviews, suggesting sufficient conceptual depth to address the study aim.

Data were collected via semi-structured interviewing on MS teams, with thematic analysis used to analyse the data. The topic guide used in the interviews was based on literature in which issues facing carers of persons living with dementia were identified. These included a systematic review on the experiences of spousal caregivers^12^. The topic guide had five sections, including an icebreaker, introductory question, before transitioning to content-related questions, and concluding with a debriefing question^29^. Example questions included:

Can you describe your experience of attending walking football sessions with the person you care for?

How has participation in walking football affected your daily life as a caregiver?

What changes, if any, have you noticed in the wellbeing of the person you care for?

Ethical approval for the study was granted by the Ethics Committee of the Institute for Health Research at the University of Bedfordshire (Reference Number IHREC996). All methods were performed in accordance with the relevant guidelines and regulations for research involving human participants. Before the interview sessions, participants were provided with a participant information sheet and the consent form. Participants were given pseudonyms to preserve anonymity. Interviews were conducted via Microsoft Teams using video to support rapport and enable observation of non-verbal communication. All interviews were audio-recorded and transcribed verbatim for analysis. All recorded videos and transcripts were password encrypted safe and destroyed after one year. Participants were informed that they could withdraw from the study at any time and that they could contact the research team whose details were present in the participant information sheet for additional details.

### Researcher reflexivity

Three researchers attended walking football sessions prior to data collection. This involvement allowed the researchers to develop contextual understanding of the activity and the participant community. However, this positioning may have influenced interpretation of the data. To address this, the research team engaged in reflexive discussions throughout the analysis process and used collaborative coding to minimise individual bias^30,31^.

### Epistemology and ontology

The study was informed by a constructivist epistemological perspective, recognising that participants’ experiences of caregiving and walking football are socially constructed and interpreted through personal meaning-making. A relativist ontology was adopted, acknowledging that multiple realities may exist across participants’ experiences^32^.

### Coding procedure

Transcripts were analysed using Braun and Clarke’s six-phase thematic analysis approach^33^. Initial coding was conducted independently by two researchers. Codes were then compared and discussed within the research team, and discrepancies were resolved through discussion until consensus was reached.

### Fidelity

Analytical rigour was supported through researcher triangulation, iterative coding discussions, and the use of verbatim quotations to support theme interpretation^34^.

## Results

Participant details are shown in Table 1. All caregivers were women, while both volunteers were men. The participants were aged 60 years or over.

**Table 1:** Demographic characteristics of study participants.

Identification Number	Pseudonym	Age	Gender	Description
WF001	Judy	69	Woman	Spousal caregiver
WF002	Layla	61	Woman	Family caregiver
WF003	Shady	65	Woman	Spousal caregiver
WF004	Robert	65	Man	Volunteer
WF005	Archer	72	Man	Volunteer
WF006	Corona	60	Woman	Volunteer/ former spousal caregiver

Five themes were identified regarding caregivers’ experiences: caregiver burden, social interaction, emotional and mental wellbeing, embodied activity and functional engagement, and quality of life. Three themes: memory recollection, physical activity and community belonging and peer support were identified for the people living with dementia. Together, these themes illustrate how walking football functioned as both a social and relational intervention that influenced caregivers’ wellbeing beyond physical activity alone. The themes were grouped by whether they were related to the caregivers (Table 2) or to people living with dementia (Table 3).

While some thematic overlap was observed, themes were delineated according to their primary analytical focus. “Emotional and mental wellbeing” captured immediate affective and psychological experiences, whereas “quality of life” reflected broader perceptions of life satisfaction, meaning, and day-to-day outlook. “Embodied activity and functional engagement” was distinguished by its focus on physical participation, capability, and activity-related functioning. This approach enabled analytical clarity while acknowledging the interconnected nature of caregivers’ experiences.

**Table 2:** Themes related to caregivers.

Theme	Quote
Caregiver Burden	“The frustration is very real. I’m sure that you’ve probably heard this from some of the others. The frustration in finding things for people to do or support is big” (Corona, WF006)
	“…I used to go into the school and listen to the readers which I liked doing but I can’t do that now because obviously I can’t leave him for a period of time” (Judy, WF001)
	“…sometimes you can’t help it, you just snap… then I feel very guilty… you carry this guilt for so long it becomes a bit of a resentment…” (Layla, WF002)
Social Interaction	“The carers that are sitting alongside, have that opportunity just to sit and chat amongst themselves… it’s valuable when you’re a carer to be able to connect with others” (Corona, WF006)
	“Walking football I think has been a saviour for a lot of people. We also do quite a lot socially… coffee after we played” (Robert, WF004)
Emotional and Mental Wellbeing	“It gives you that little break away from the house and the housework can stay for another day… you’re not thinking about caring at that time” (Shady, WF003)
	“We sit and talk and laugh about it because if you don’t laugh, you’ll cry” (Layla, WF002)
	“It gives them a social respite” (Robert, WF004)
Embodied Activity and Functional Engagement	“It just gives me exercise that I don’t normally do… it gets the heart going, which is very important” (Judy, WF001)
	“If I play, I really feel the benefits of it… it’s good for carers as well…” (Layla, WF002)
	“It definitely upped my level of exercise, which then pushed me to join a gym” (Corona, WF006)
Quality of Life	“Participating in exercise, socializing with like-minded people… seeing there is a life after it” (Judy, WF001)
	“Before walking football, there wasn’t really much to look forward to… there’s something to look forward to now for him and for me” (Layla, WF002)
	“…I only played when my husband was alive… but yes, thoroughly enjoy it… see the benefits of [WF]” (Corona, WF006)

The themes presented in Table 2 illustrate the complex and interconnected nature of caregivers’ experiences. Caregiver burden was characterised by emotional strain, restricted personal time, and persistent feelings of guilt. Participation in walking football created opportunities for social interaction and respite, enabling caregivers to step temporarily outside the caregiving role. Physical engagement further contributed to a sense of capability and self-maintenance, while these combined experiences appeared to underpin broader improvements in perceived quality of life.

**Table 3:** Themes related to PLWD.

Theme	Representative Quote(s)
1. Memory Recollection	“…and then you give some of them a ball and they change. It’s almost like turning the clock back.” (Robert, WF004) “…give them a ball and you can see that time go back. It’s brilliant to watch.” (Corona, WF006) “He was always out, always on the playing field…even now in these later stages of dementia, you can imagine when he was tickety boo how good he was.” (Layla, WF002) “…must have been an excellent footballer in his youth because…he’s still got physical moves…” (Archer, WF005)
2. Physical Activity	“…but what this has made him realize is that he can do it, and he can play football for that period of time.” (Judy, WF001) “…he gets plenty of exercise which he needs.” (Layla, WF002) “I think it kept him more able to move about while he was still able to do the football…” (Corona, WF006) “…it’s keeping what they’re capable of, still able to do… in their routine and it helps to keep them fit.” (Corona, WF006) “…I had helped through WF to help maintain their level of PA…” (Archer, WF005)
3. Community Belonging and Peer Support	“A lot of them…will say it’s the highlight of their week.” (Robert, WF004) “…coffee time is an important time…to be able to talk with their husbands there…involve them in a big round table conversation.” (Archer, WF005) “…we all join in together, have a cup of tea and a piece of cake and then they’re all happy.” (Shady, WF003) “…he found the company of the others around him as well was good.” (Shady, WF003)

The themes relating to people living with dementia show that walking football supported cognitive, physical, and social engagement. Participation appeared to prompt moments of memory recollection, maintain physical activity and functional ability, and foster a sense of inclusion through shared routines and peer interaction. Together, these findings suggest that walking football offered benefits extending beyond exercise alone.

## Discussion

The purpose of this study is to examine the perspectives of caregivers who engage in an activity of walking football with persons living with dementia. The findings of this study show that the activity of walking football provides caregivers with benefits that transcend the level of physical activity, providing them with a relational intervention that alters their caregiving experiences, identities, and emotions. Past studies have shown that persons living with dementia benefit from various positive aspects of sports-based activities, although this study provides additional insight to the benefits of these activities, especially through the experiences of caregivers. This study should therefore be understood as exploratory, offering context-specific insight rather than claims of broad transferability.

The most significant finding of this study is that walking football functioned as a form of co-participatory respite, allowing caregivers and people living with dementia to engage in shared activity rather than reinforcing traditional caregiver–recipient roles. This relational dynamic appeared to support both emotional wellbeing and social connectedness for caregivers.

Walking football enabled the co-enjoyment of the activity and relational continuity, such that the caregiver and care recipients were able to co-participate in the activity of walking football instead of the traditional binary of “carer” and “cared-for.” This finding mirrors the relational approach to dementia care, which seeks to ensure the personhood of the individual with dementia by connecting with others and finding meaning^27^. Unlike traditional respite care settings, which can separate caregivers and care recipients, walking football was co-present respite care. This was particularly relevant, given that guilt was identified by Quinn et al.^35^ as a barrier to respite care for caregivers. Guilt was also identified by Brodaty and Donkin^36^ as a barrier to caregivers accessing respite.

Furthermore, benefits to caregiver emotional health, which encompassed stress reduction, positive mood, and normalcy, have also been identified. The above findings support existing literature which argues, among other factors, caregiver emotional health is significantly associated with social engagement and support opportunities, and identity^37^. Walking football seemed to facilitate an environment where caregivers might escape the role of caring for a moment and reconnect with elements of their pre-care identity, and interact socially with others who shared the same lived experience. Notably, the social interaction occurred without the need for support infrastructure, which some caregivers find stigmatising and emotionally draining^38^.

Finally, the social aspect of walking football was prominent. The caregivers appreciated the feeling of belonging and community created by regular participation, which aligns with previous research on group-based physical activity and social prescribing^39,40^. The importance of community is of particular significance to dementia care, especially due to the level of social isolation that caregivers and care recipients experience^41^. The informal and familiar football environment was found to minimize participation barriers, especially for the male participants, corroborating previous studies on gender and culturally specific activities for dementia care^42^.

Another important contribution that this research has made lies within the challenge that it poses to the “deficit model” that often surrounds the experience of caregiving. Indeed, the experience of caregiving can often be defined within the context of burden or strain; yet the participants also spoke about the experience of shared pleasure. This supports the emerging literature on “caregiver gain,” where the experience of caregiving can include not only negative elements but also the positives. Walking football could contribute to this gain through the experience of shared success. The findings highlight the coexistence of caregiver burden and caregiver gain. While participants described emotional strain, frustration, and guilt associated with caregiving, walking football created opportunities for shared enjoyment, social belonging, and identity reconstruction. This duality reflects emerging literature suggesting caregiving experiences are not solely deficit-based but may also include positive meaning-making.

From an applied perspective, the results imply that walking football can be considered not only as an ‘activity’ but as an affordable community-based ‘psychosocial intervention’ to facilitate the needs of caregivers as much as those suffering from dementia. This aligns well with the ‘public health priorities’ around ‘age well’, ‘dementia friendly communities’, and ‘social prescribing’^43,44^. Walking football can be considered as an opportunity to address the ‘unmet need’ in caregiver interventions, as much as it can facilitate ‘physical activity’ and ‘social engagement’.

### Limitations and future research

However, there are some limitations that need to be addressed. For example, this study used a small number of participants, all of whom were part of a walking football programme in the UK. In addition, this study used self-selecting participants, which may be biased towards those caregivers who are already involved in community activities. There were also more women than men, which, although reflective of general demographics, prevented further analysis of gender differences in terms of caregivers. Additionally, the small sample size and limited demographic diversity restrict the transferability of the findings. Participants were predominantly older caregivers involved in a single community programme, and experiences may differ across cultural contexts or caregiving arrangements.

Future studies should aim to recruit more diverse participants, as well as undertake longitudinal and comparative studies to further analyse how walking football differs from other forms of activity or respite.

## Conclusion

This study shows that there are significant benefits for caregivers that can be derived from playing walking football, such as promoting relational wellbeing, alleviating emotional pressure, and encouraging sociability. This study contributes to the current literature around sport for health and dementia care, which emphasises the importance of interventions that meet the needs of both caregivers and care receivers jointly. With the rise of dementia, such relationally focused, community-based interventions should be recognised more prominently.

## Data Availability

The data supporting the findings of this study are not publicly available due to ethical restrictions related to participant confidentiality. Anonymised excerpts are included in the article. Further information may be available from the corresponding author upon reasonable request.
